# DCPVP: Distributed Clustering Protocol Using Voting and Priority for Wireless Sensor Networks

**DOI:** 10.3390/s150305763

**Published:** 2015-03-10

**Authors:** Hooman Hematkhah, Yousef S. Kavian

**Affiliations:** Faculty of Engineering, Shahid Chamran University of Ahvaz, Ahvaz 61639, Iran; E-Mail: hooman.hematkhah@gmail.com

**Keywords:** wireless sensor network, clustering, routing, voting, prioritize, energy efficiency

## Abstract

This paper presents a new clustering protocol for designing energy-efficient hierarchical wireless sensor networks (WSNs) by dividing the distributed sensor network into virtual sensor groups to satisfy the scalability and prolong the network lifetime in large-scale applications. The proposed approach is a distributed clustering protocol called DCPVP, which is based on voting and priority ideas. In the DCPVP protocol, the size of clusters is based on the distance of nodes from the data link such as base station (BS) and the local node density. The cluster heads are elected based on the mean distance from neighbors, remaining energy and the times of being elected as cluster head. The performance of the DCPVP protocol is compared with some well-known clustering protocols in literature such as the LEACH, HEED, WCA, GCMRA and TCAC protocols. The simulation results confirm that the prioritizing- and voting-based election ideas decrease the construction time and the energy consumption of clustering progress in sensor networks and consequently improve the lifetime of networks with limited resources and battery powered nodes in harsh and inaccessible environments.

## 1. Introduction

The use of wireless sensor networks (WSNs) has grown enormously due to the great variety and capability of their modern and real-world applications [1] in areas such as e-health, space and extreme environment applications, traffic control, smart home applications and object tracking [2]. A typical wireless sensor network is made up of some sensor nodes which are distributed in the region of interest (ROI) which are often harsh and inaccessible environments. The sensor nodes collect data from the environment in the proposed application and forward that to a data link using single-hop and multi-hop communication schemes. The sensor nodes are battery powered and it is usually impossible to recharge or replace the battery under harsh conditions. That is the main constraint for designing and employing such sensor networks with limited energy nodes in large-scale applications [3,4].

There are some ideas and suggestions for decreasing the energy consumption in different network layers such as using low power sensors and energy management of local processors in physical layer and other methods to improve the lifetime of the whole network instead of a single node, such as MAC protocols in data link layers [5], routing protocols in the network layer [6], the network coding [7,8,9] and retaining the network connectivity approaches [10,11].

Clustering is one of the most important methods for designing a hierarchical energy efficient sensor network to achieve reliable data transmission, scalability and load-balancing criteria [12]. In clustering schemes, sensor nodes are grouped into virtual groups with a cluster head (CH), and other sensor members. The members measure physical parameters and send them to the cluster head and the cluster head is responsible for forwarding the aggregated data to other cluster heads or to the base station [12,13].

Clustering protocols use different parameters such as the energy levels of nodes, the node density and centrality for network virtual portioning and selecting cluster heads [14] and some other ideas for robustness against the network topological changes [15]. As in monitoring applications of WSNs, the data flow direction is from sensor nodes to the BS, therefore every node delivers its generated data to the base station somehow. The routing between CHs for forwarding data can be either direct or multi-hop. The direct routing approach is easy to implement and fast in delivery, so it is used in many cases, but when the size of sensor network grows, this method can be inefficient or useless, so in order to improve the protocol scalability, intercluster routing by multi-hop schemes are employed [15,16,17] which are also considered in this paper.

In multi-hop routing schemes, the cluster heads that are closer to the base station have the responsibility of forwarding data packets of all other cluster nodes to the data link, so an important parameter for clustering and the cluster size is the distance from the base station where the size of clusters closer to the base station is smaller than that for farther clusters. This is an efficient way to avoid hot nodes dying [17,18] and is considered in this paper. The aim of this paper is to present a new clustering approach which is based on voting and priority ideas and called the DCPVP protocol.

The remainder of this paper is organized as follows: Section 2 describes the previous related works and explains the LEACH, HEED, WCA, GCMRA and TCAC protocols. Section 3 presents the network and energy models and assumptions. Section 4 describes the proposed protocol in detail and explains the different phases of the DCPVP protocol. A simulation test-bench including the assumptions, parameters and scenarios are presented in Section 5. Finally, Section 6 concludes the paper.

## 2. Related Works

### 2.1. The LEACH Protocol

The Low-Energy Adaptive Clustering Hierarchy (LEACH) protocol [19] proposed by Heinzelman *et al.*, is one of the basic clustering and routing protocols in WSNs and is used by many subsequent clustering and routing protocols. The main idea of LEACH is to select cluster heads by rotation and the high energy consumption for communicating with the BS is spreads among all the nodes.

The operation of LEACH consists of rounds, and each round consists of two phases; the set-up phase and the steady-state phase. In the set-up phase the clusters are formed and in the steady-state phase data is delivered to the BS. In the set-up phase, each node decides to become a CH or not for the current round. The decision is based on the suggested percentage of CHs for the network and the times of being CH so far. The node generates a random number between 0 and 1, then it becomes a CH for the current round if the number is lower than the threshold *T*(*i*), as follows:

(1) $$T\left(i\right)=\{\begin{array}{c} \frac{P}{1-P(r\;mod\frac{1}{P})}\;,\;if\;n\in G\\ \;0\;,\;otherwise \end{array}$$

where *P* is the desired percentage of CHs, *r* is the number of current round, and *G* is the set of nodes that have not been elected as CHs in the last *1/P* rounds [16]. When a node is elected as CH, it broadcasts an advertisement packet. According to the received signal strength, other nodes decide which cluster they could join [20,21].

### 2.2. The HEED Protocol

The Hybrid Energy-Efficient Distributed Clustering (HEED) protocol [22], proposed by Younis and Fahmy, is a multi-hop clustering protocol which provides energy-efficient clustering. Unlike the LEACH protocol that randomly selects nodes as CHs, the HEED selects the CHs based on residual energy and intra-cluster communication cost. One of the main ideas of HEED is to achieve a balanced distribution of CHs throughout the network. Moreover, the probability of two nodes within each other’s communication range becoming CHs at the same time is very small in the HEED protocol. Initially, *C_prob_*, a percentage of CHs among all nodes, is set to assume that an optimal percentage cannot be computed. The probability of which a node becomes a CH is:

(2) $$CH_{prob}=C_{prob}\frac{E_{residual}}{E_{max}}$$

where $E_{residual}$ is the estimated current energy of the node, and $E_{max}$ is a reference maximum energy, which is equal for all nodes. The value of $CH_{prob}$ is not allowed to be less than a certain threshold and the threshold is inversely proportional to $E_{max}$. After that, each node executes several iterations to find the CH. On the other hand, CHs forward data to the BS using a multi-hop communication scheme [23].

### 2.3. The WCA Protocol

The Weighted Clustering protocol [24] proposed by Chaterjee *et al.* is based on nodes’ neighbors’ number and it considers the movement of nodes. The CH election is based on node degree (number of neighbors), transmit and receive energy and residual energy. To ensure that CHs is would not be under overload or high energy consumption conditions, there is a threshold number which shows the maximum number of cluster members. In other word the cluster size is limited [25,26]. This fact that CH election process does not happen periodic causes reduction in calculations. The nodes would be elected as CH according to their weight which is:

(3) Wv = W1 × Δv + W2 × Dv +W3 × Mv +W4 × Pv

where v is the ID of node, Δv is obtained by subtracting the threshold from the number of the neighbors, Dv is summation of distances of v node from all its neighbors, Pv represents consumed energy and Mv indicates the mobility. The node with minimum weight is elected as CH. After that this process iterates until each node either finds a cluster or becomes a CH.

### 2.4. The GCMRA Protocol

The Energy efficient Grid based Clustering and Routing Protocol [27], proposed by Jana and Jannu, is a location-based method that divides the whole region into several grids. Nodes in every grid form a cluster. After the cluster forming step, cluster members elect the most suitable node as CH. According to the transmission range of nodes and considering the fact that every node in each cluster should be able to communicate with every node in eight-neighbor clusters, the grid size is calculated as *R = x/2.83*. On the other hand, the number of clusters can be calculated by knowing the grid and the network size, so the number of clusters in this method is fixed.

After finding the clusters, the nodes start by calculating the sum of distances from all nodes in a cluster. Finally the node with a minimum sum of distances becomes CH as long as its energy level is higher than a set threshold. This protocol uses a multi-hop routing scheme between CHs for shorter transmissions. As the relay nodes are between the source cluster and the BS, first, all nodes consider the BS as next-hop and if there is a CH in its radio range that is closer to BS, it becomes the next-hop. In fact, this approach focuses on reduction the communication range, and consequently reduction in long distance communication energy consumption [27].

### 2.5. The TCAC Protocol

The Topology-Controlled Adaptive Clustering (TCAC) protocol [28] was proposed by Dahnil *et al.* In this protocol all clustering steps are done assuming that the transmission energy of the nodes can vary. This method has three phases; the first is a periodic update. In order to reduce the effect of energy overhead (transmission start-up cost) and delay time, the periodic update is executed once in every *D* cycles. If this process were to execute in every cycle, the delay time and energy consumption would increase. In second phase, which is CH election phase, every node generates a random number between 0 and 1 and compares it with *P*(*CCH*). If the random number is less than *P*(*CCH*), the node becomes a candidate where the *P*(*CCH*) is the probability of becoming a candidate that is calculated as the ratio of residual energy of node and the average energy of all network nodes. After electing the candidates, the competition between them starts. The candidate with the most energy among all the candidate neighbors becomes the CH. In third phase, the CHs send a packet and each node that receives the packet responds it with another packet. Afterward the CH creates and broadcasts a list of nodes that send the packet and rank them based on the signal strength. Nodes use the list to find the best CH. This protocol focuses on the scalability of the network. In other word, increasing the number of nodes doesn’t affect the efficiency of this protocol [28].

## 3. Network and Energy Models and Assumptions

### 3.1. Network Model and Assumptions

The network model is considered as a graph *G* = (*V*, *E*), where *V* is a set of nodes which contains the BS and N sensor nodes distributed in the ROI. The BS node has an unlimited energy source and it can be placed inside or outside of the desired region and collects the receiving data. *E* ⊆ *V^2^* is the set of links, if two nodes are in communication range of each other, there is a link between them. The links are symmetric and bidirectional. Since nodes do not use GPS positioning equipment, they are not aware of their own geographical coordinates. The nodes equipped with RSSI to measure their distances. The nodes are similar and are distributed evenly or randomly in a square-shaped field. Nodes explore the network topology independently. They send data packets with fixed signal strength and on a fixed frequency. Nodes’ position is assumed fixed and the initial energy of nodes is assumed similar.

### 3.2. The Energy Model

**Figure 1:**
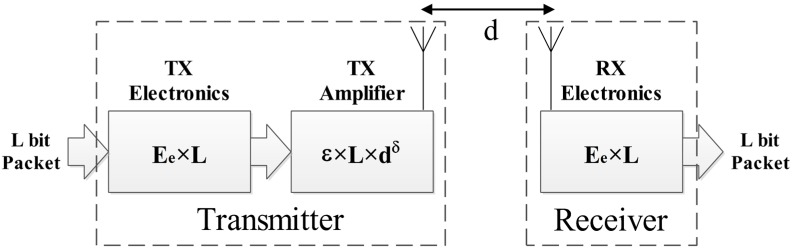
The transmission and receiving energy consumption model.

The same radio-energy model as stated in [29] is used, which is described briefly as follows. The schematic of the model is presented in Figure 1. This model considers the transmission energy in two parts. The first part is the amplification energy (propagation loss) that depends on the number of bits, the distance from the receiver and the acceptable bit-error rate. The propagation loss is proportional to $d^{2}$ for distances less than $d_{0}$ and is proportional to $d^{4}$ for distances more than $d_{0}$.

For the receiving energy model, only the electronic processing energy, that depends on the number of bits, is considered. For L*-*bit packets over a distance d, the consumed energy is:

(4) $$E_{Tx}(L,d)\;=\;E_{Tx-e}\;(L)\;+\;E_{Tx-amp}(L,d)$$

(5) $$\;E_{Tx}\left(L,d\right)=\{\begin{array}{c} \;L.E_{e}\;+\;L.\varepsilon_{fs}.d^{2}\;,\;d\leq d_{0}\\ \;L.E_{e}\;+\;L.\varepsilon_{mp}.d^{4}\;,\;d>d_{0} \end{array}$$

(6) $$E_{Rx}\left(L\right)\;=\;E_{Rx-e}\;\left(L\right)=L.E_{e}$$

where $E_{Tx}$ is transmitter energy consumption, $E_{Rx}$ is the receiver energy consumption, $E_{Tx-e}$ and $E_{Rx-e}$ are the electronic processing energy consumption and $E_{Tx-amp}$ is the amplifier energy consumption.

## 4. The DCPVP Protocol

**Figure 2:**
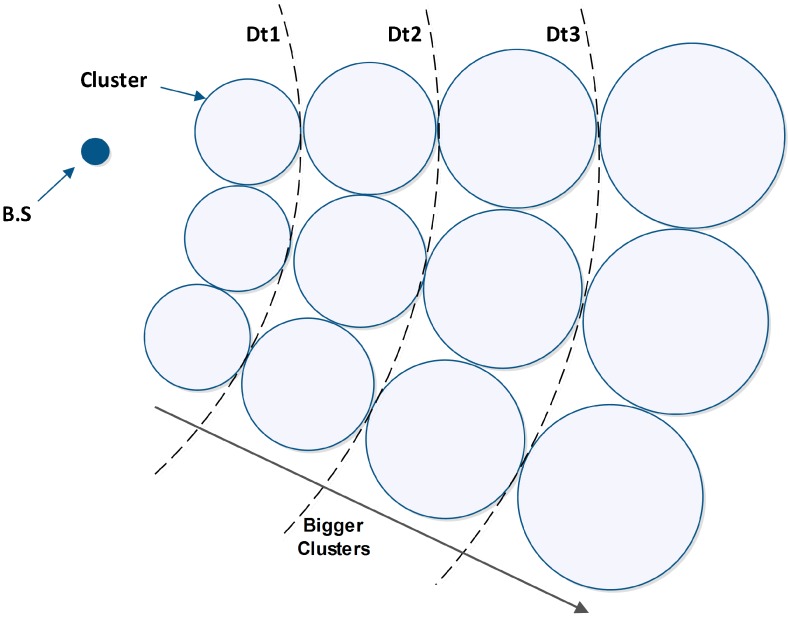
The cluster size is proportional to distance from BS.

In the previous protocols, such as LEACH and HEED, the size of clusters is uniform regardless of the distance from the BS, which causes early death of some nodes. Since the energy consumption increases proportionally to transmission distance, multi-hop routing is used by CHs, so the neighbors of a BS have the duty of forwarding data packets of farther nodes to the BS. On the other hand, as the cluster size increases, the CH energy consumption increases too. According to the discussed issues, we assume the size of clusters is proportional to the distance from BS, which provides balancing of CHs’ lifetime in different places. This means that closer clusters to the BS have smaller sizes. To avoid uncontrolled increases in the cluster number, as the distance from the BS increases, the cluster size will be bigger [30], as shown in Figure 2.

**Figure 3:**
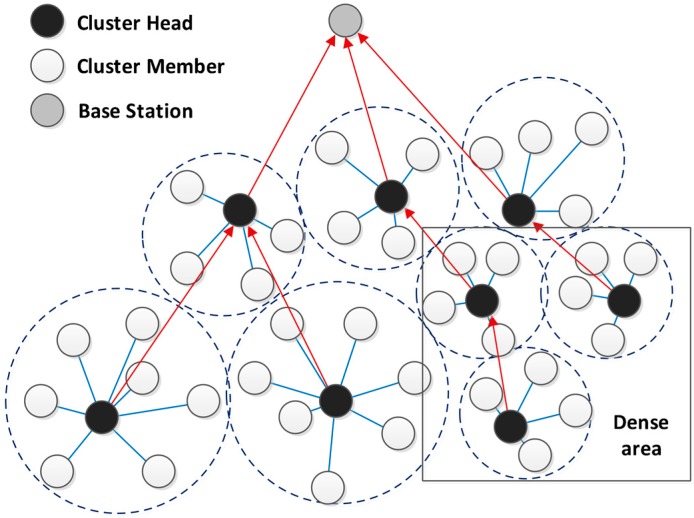
The performance of DCPVP in dense areas.

On the other hand, in the places where nodes are distributed densely, by choosing one node as the CH, the energy consumption of that node increases extensively and may cause its death. To avoid that, the size of clusters should be smaller and the number of them should increase in such places. Therefore, the load of several nodes is prorated over several CHs and this avoids the death of a single node [30] (Figure 3).

As mentioned, in the DCPVP method, nodes have similar roles in the clustering process, so the control manner of this protocol is distributed, which causes the protocol to be scalable and makes it more adaptive. However, in centralized methods by increasing the number of nodes, the manager node should be able to access all nodes and in some cases this is not possible. On the other hand, as the number of nodes increases, the time of the clustering process increases too and relative to steady state cycle times this becomes inefficient. According to all these issues we can mention that the main idea of DCPVP is choosing the cluster size based on the nodes’ distance from the BS, the local density of nodes and the nodes’ average distance from neighbors. We should mention that the important parameters such as residual energy times being elected as CH are also considered. The protocol includes five phases which are described in details.

### 4.1. Phase A: Exploration Phase

In this phase, each node explores the network topology and gathers some information which includes the node distance from the BS, the number, and distance from its own neighbors. Distances are calculated by the RSSI equation [31,32]:

(7) $$d(v)\;=\;10^{\frac{\left|RSSI-RSSI_{0}\right|}{10\;.\;\;\eta }}$$

where *RSSI* is the received signal strength, *RSSI_0_* is the signal attenuation for one meter distance from the source node and *η* is the path loss exponent [33].

When the exploration phase begins, the BS broadcasts a packet (Start-Packet) to inform nodes of the beginning time and to let nodes calculate their distances from the BS. Then every node sequentially (based on ID) sends a packet to its neighbors during its own time slot (Hello-Packet) which contains its ID. All the other nodes monitor the channel during this time slot, so the neighbor nodes can receive the packet and store the information of the packet and the distance which is measured by RSSI in their local memory (Table 1). The nodes’ information about the network topology depends on the neighbors ID, so if a node dies, the neighbor node should only know its ID and modify its calculations as the network is considered as being stationary. This phase is a significant preparation for the next phases and is done once for a network. After the exploration phase, DCPVP operates in rounds. In every round, the clusters’ construction should be rebuilt and new CHs should be elected. After clustering, nodes generate Data-Packets and the network works normally, so the remaining phases repeat in every round. At the end of this phase, each node forms a table such as Table 1, and then updates and uses it later.

**Table 1:** Primary nodes’ information from the network topology.

Neighbors	ID	Distance (m)
1	32	0.56
2	57	4.41
...	...	...
M	4	3.1

**Figure 4:**
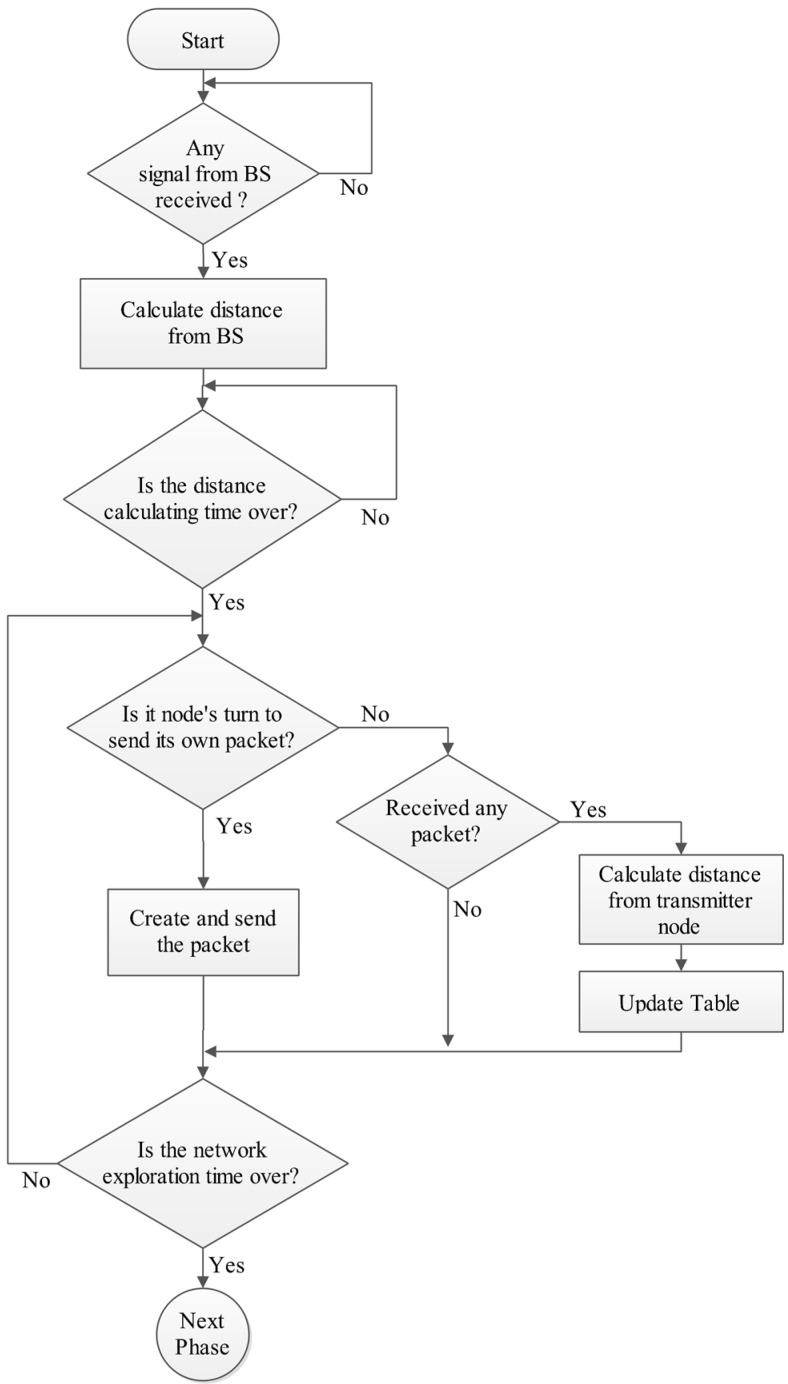
Phase A: Exploration phase.

Table 1 is updated only when a node dies in its neighborhood. When the node’s energy level falls to less than a predefined threshold, the node is considered dead and it notifies its neighbors by sending a corresponding packet (Death-Packet). Every node that is in its neighborhood receives the packet and removes its information row from the Table 1. Figure 4 shows the flowchart of this phase.

### 4.2. Phase B: Cluster Head Election Phase

In this phase, each node calculates its own weight:

(8) $$W\;=\;\alpha \;F(\text{MD})\;+\;\beta \;F(\Delta C)\;+\;\gamma \;F(\text{Y})\;-\;\phi \;F(\frac{E_{res}}{E_{0}}),\;0\leq \alpha ,\beta ,\gamma ,\phi \;\leq 1$$

where α, β, γ and ϕ are adjust coefficients, Y is the times that the node has been a CH so far, $E_{res}$ is the residual energy, $E_{0}$ is initial energy, MD is mean distance to neighbors and ΔC is the optimum deviation. MD can be calculated as follows:

(9) $$MD\;=\frac{\sum_{i=1}^{\text{Ns}}\text{DV}(\text{i},\text{x})}{N_{s}}$$

where X is the nodes’ ID, $N_{s}$ is the number of neighbors, DV is the distance vector, DV (i, j) is the distance between the nodes i and j and ΔC can be calculated as follows:

(10) $$\Delta C\;=\left|\;N_{s}\;-\;N_{O}\;\right|$$

where $N_{s}$ is the number of neighbors and $N_{O}$ is optimum number of the neighbors (See Figure 2):

(11) $$N_{O}\;=\;floor\;\left(N_{r}\right)$$

(12) $$N_{r}\;=\;\{\begin{array}{c} \;\;1\;\times \;Nm\;\;\;\\ C1\;\times Nm\\ C2\;\times Nm\\ C3\;\times Nm \end{array}\;\;\;\begin{array}{c} \;\;\;\;\;\;\;\;\;\;\;\;\;D>Dt1\\ Dt1>D>Dt2\\ Dt2>D>Dt3\\ \;\;\;\;\;Dt3>D \end{array}\;,\;0<C3<C2<C1<1$$

where D is the distance from BS, Nm is maximum cluster size, Dt1, Dt2 and Dt3 are threshold values of distance and C1, C2 and C3 are the coefficients and are less than 1 (C1, C2, C3 < 1). Thus we allow the nodes which are farther from BS to form bigger clusters, and allow the closer nodes to only form smaller clusters. This allows load balancing. In this step, each node calculates its own weight and broadcasts it sequentially. Now each node adds a column to its table and writes the weight of each neighbor in it as Table 2.

**Table 2:** Nodes information from network topology in every round.

Neighbors	ID	Distance (m)	Weight
1	32	0.56	W_1_
2	57	4.41	W_2_
…	…	...	...
M	4	3.1	W_m_

**Figure 5:**
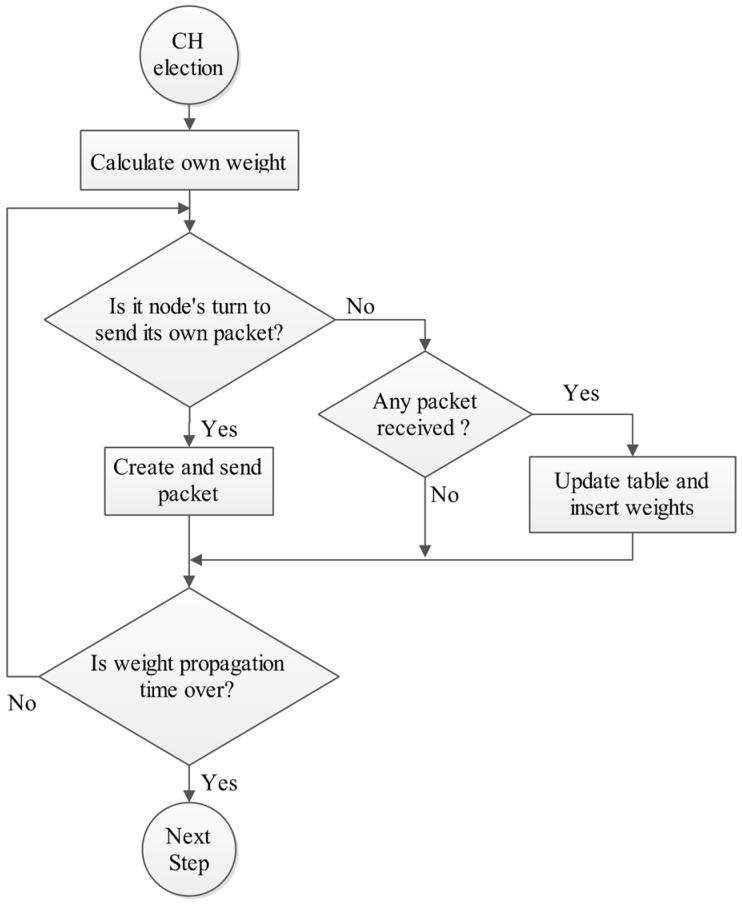
Phase B: Cluster head election phase, Step 1.

**Figure 6:**
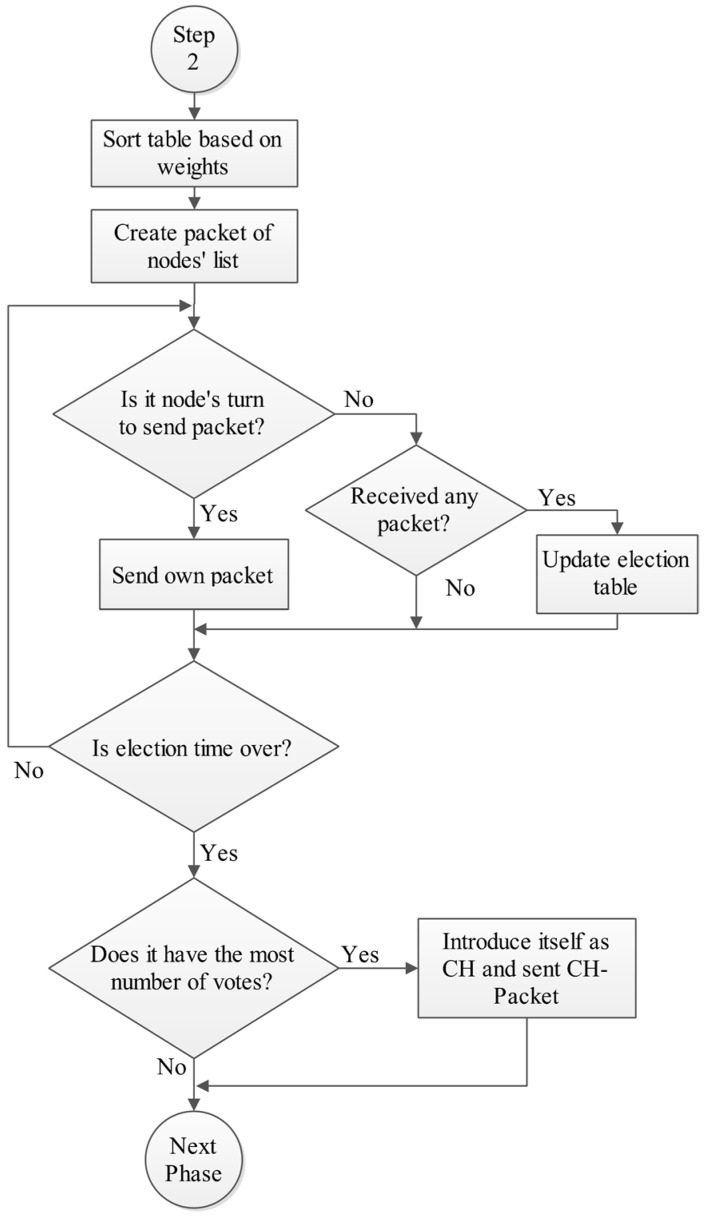
Phase B: Cluster head election phase, Step 2.

Then each node creates a priority list from its neighbors based on their weight. After that they broadcast a packet containing the voting list (Vote-Packet) and vote the best nodes. In other words, the Vote-Packet is a list of nodes’ IDs which are sorted based on their weight. After finishing the election, the node which has the most number of votes, is chosen as the CH and introduces itself by sending a packet (CH-Packet). Figure 5 and Figure 6 show the flowchart of this phase.

All ID-based sequential broadcasts have the same following algorithm. When node i wants to send its own packet, it waits for i-1 time slots from the end of last step until its turn comes and then sends its own packet. During all other time slots the node monitors the channel for receiving probable packets [33].

### 4.3. Phase C: Cluster Building Phase

As discussed before, the CH introduces itself by sending a packet to its neighbors. All nodes in its radio range hear the packets and are aware of the voting result, so when they receive the CH-Packet they respond by sending a packet to the CH (Join-Packet). The CH accepts their join request based on their weight from less to greater. In other words, the N_O_ accepted nodes in cluster are the N_O_ lower nodes in the weight-sorted table. The reason for priority voting is that often, after primary clustering, there are some nodes that are not in any cluster, because of the limited size of clusters, so in a second step, the nodes which have the highest vote and were not elected in the previous step introduce themselves as CH and the previous process will repeat. Finally, if there is a node which is neither organized nor received any packets, it introduces itself as a CH (outlying nodes). The benefit of this iterative method is that we don’t have overlapping clusters. For example when node Y becomes a CH in a specific region it certainly has received the highest number of votes, which means that all neighbor nodes know Y as the most weighted node. The elected CH forms a cluster and accepts nearby nodes into its cluster. Also in some regions where the density of nodes is high and the cluster size is limited, the cluster is filled and some nodes will not be organized. In this case according to weight-based acceptance, the remaining nodes are the high weighted nodes of the previous step. These nodes elect the most weighted node among themselves as CH. Finally, after organizing all the nodes, the elected CHs form a timing table for their cluster members and the network goes into steady-state phase [34].

### 4.4. Phase D: Cluster Head Routing Phase

**Figure 7:**
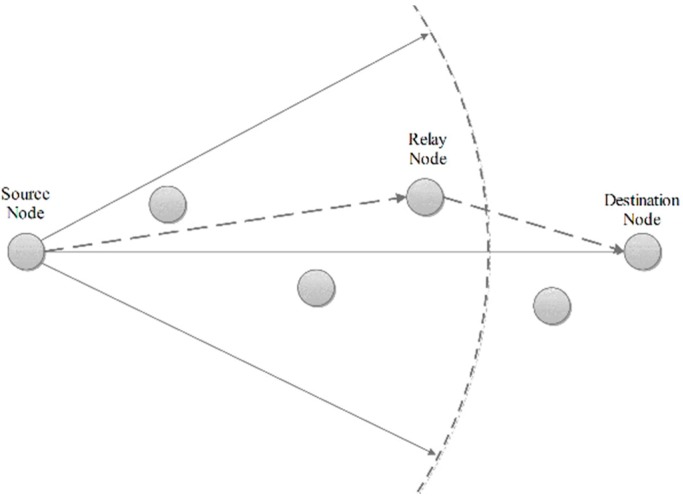
The cluster head routing phase.

**Figure 8:**
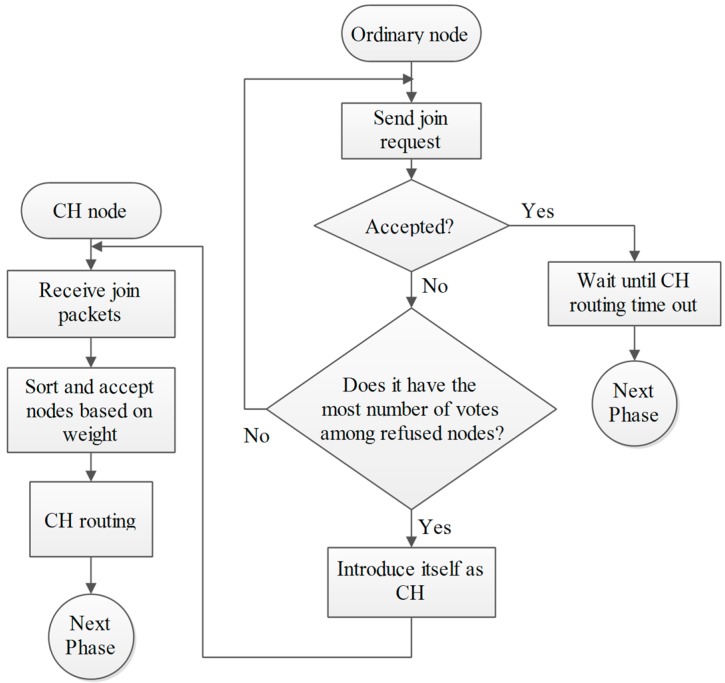
Phase C and D.

Data forwarding to the BS is multi-hop and done by other CHs. Routing between CHs is done before the steady state phase using the “Most Forwarding Progress within Radius” technique (Figure 7). CHs implement this technique by knowing their distance to the BS and share it with other CHs. Any CH transmits its data to the CH which is closer to the BS and is in its radius. The flowchart of phases C and D is shown in Figure 8.

### 4.5. Phase E: The Steady State Phase

In this phase, the network works normally and nodes sense the desired environmental parameters and transmit them to the BS. This phase continues until a certain time (t_C_), which varies depending on the application. Then, the above cycle from the phase B repeats. In this protocol, each node has the chance to be elected as the CH. The optimum results are obtained by tuning the adjust coefficients.

## 5. The Simulation Results

In simulation experiments, the sensor nodes are distributed in a 50 m × 50 m area and the BS is located at (100, 25). Initial values are summarized in Table 3. The MATLAB tool is employed for providing simulation test-bench. The results are compared with the results of previous protocols such as the LEACH, HEED, WCA, GCMRA and TCAC protocols. For a fair comparison, when 80% of nodes die, the network will become useless which is considered in all protocols.

**Table 3:** The initial values.

Pararameter	Description	Value
E (J)	Initial energy of one node	0.5
N_m_	Maximum allowed Cluster Size	10
C1	Coefficient	0.8
C2	Coefficient	0.6
C3	Coefficient	0.4
Dt1 (m)	Distance threshold	1 × Xmax
Dt2 (m)	Distance threshold	0.7 × Xmax
Dt3 (m)	Distance threshold	0.4 × Xmax

**Figure 9:**
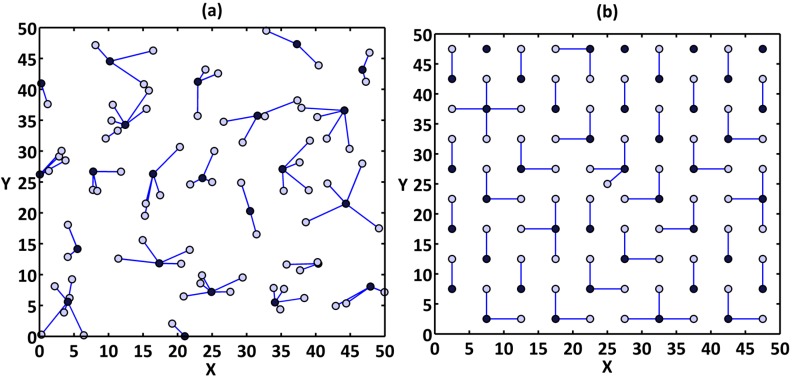
The network clusters for 100 nodes; (**a**) distributed randomly; (**b**) evenly.

The number of nodes for random cases is 100, 125, 150, 175, 200, 225 and 250 nodes and for uniform cases are 100, 121, 169, 196 and 225 nodes. All these cases are simulated for all protocols. Figure 9a,b shows the network clusters provided by the DCPVP protocol for 100 nodes in random and uniform distribution, respectively.

**Figure 10:**
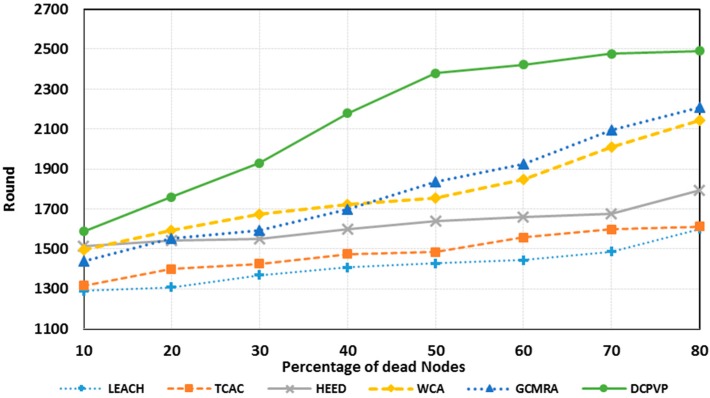
The percentage of dead nodes in uniform distribution for 100 nodes.

**Figure 11:**
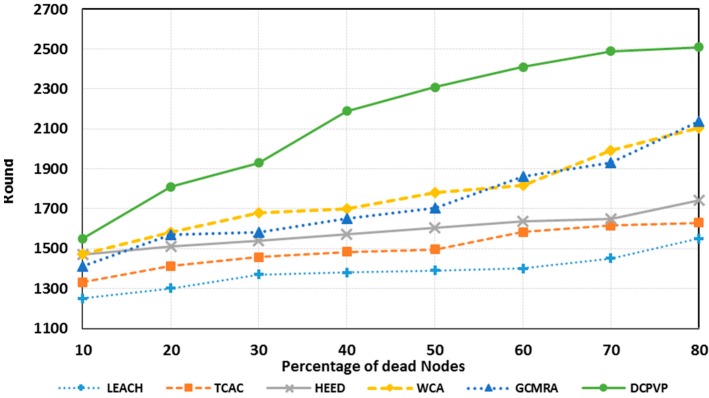
The percentage of dead nodes in uniform distribution for 144 nodes.

**Figure 12:**
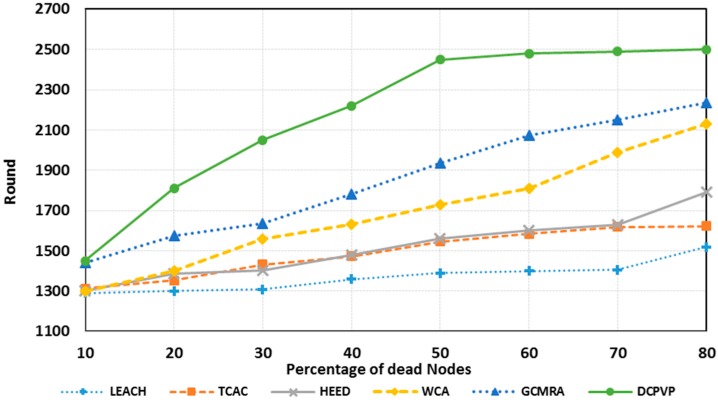
The percentage of dead nodes in uniform distribution for 196 node.

The percentage of dead nodes in uniform distribution for all protocols is presented for 100 nodes in Figure 10, for 144 nodes in Figure 11 and for 196 nodes in Figure 12. As shown in Figure 10, Figure 11 and Figure 12, for the equal round number in uniform distribution, the percentage of dead nodes in DCPVP is less than the other protocols.

**Figure 13:**
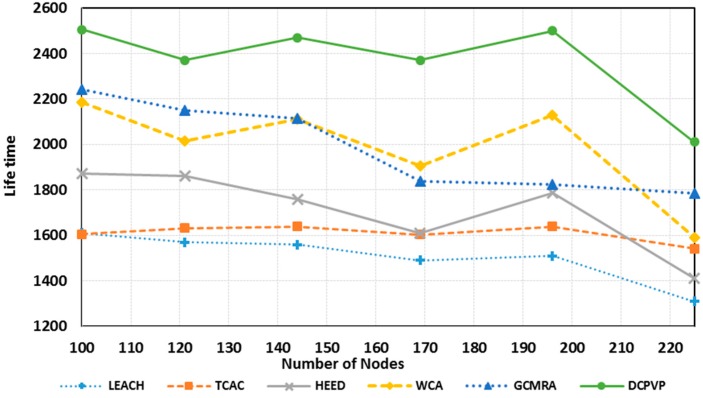
Comparison of the network lifetime in uniform distribution.

Figure 13 shows the network life-time in 20 simulation experiments *versus* the number of nodes in uniform distribution until the network becomes useless [30]. Compared to other protocols, the DCPVP protocol shows better performance for all experiments and after increasing the number of nodes this protocol still performs better than all other protocols.

**Figure 14:**
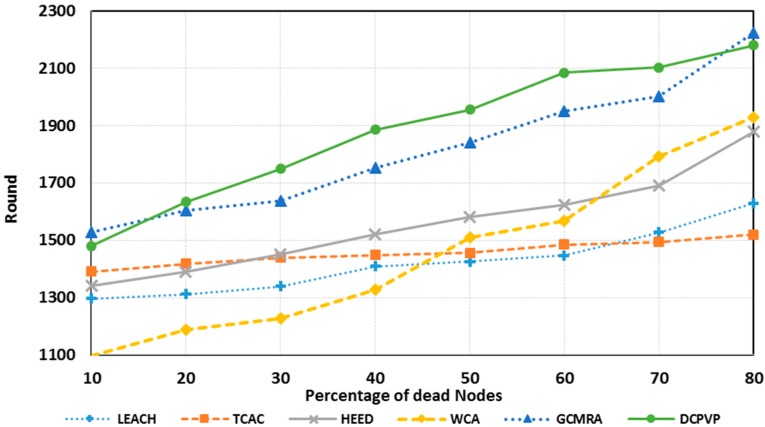
The percentage of dead nodes in random distribution for 100 nodes.

**Figure 15:**
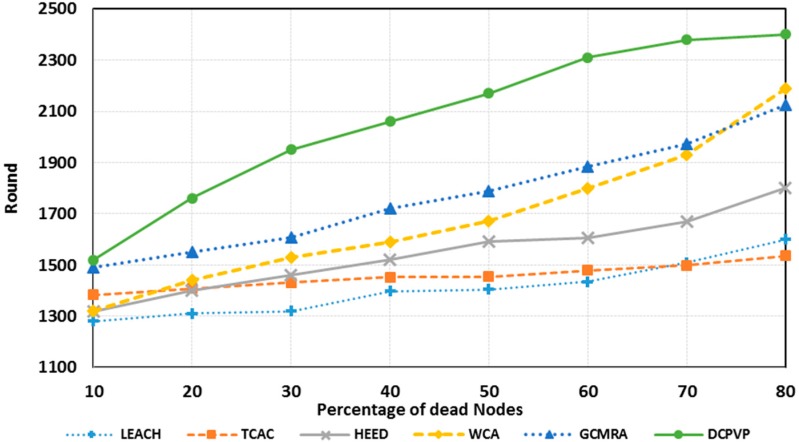
The percentage of dead nodes in random distribution for 150 nodes.

**Figure 16:**
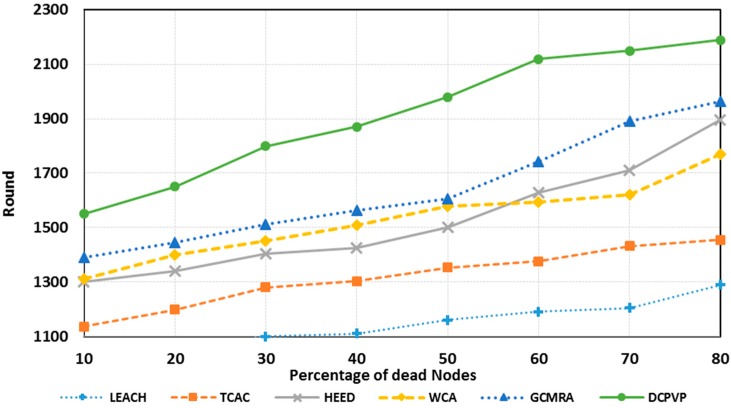
The percentage of dead nodes in random distribution for 250 nodes.

**Figure 17:**
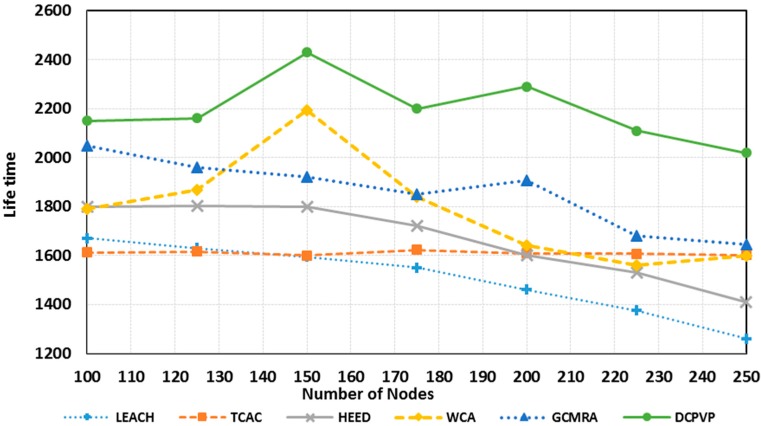
Comparison of lifetime in random distribution.

The number of dead nodes for all protocols is presented in Figure 14 for 100 randomly distributed nodes, for 150 randomly distributed nodes in Figure 15 and for 250 randomly distributed nodes in Figure 16, respectively. As shown in Figure 14, Figure 15 and Figure 16 for an equal number of rounds, the percent of dead nodes in the DCPVP protocol is ostensibly less than in the other protocols. Furthermore, in some cases when just 10% of nodes in DCPVP die, the networks life time is finished in some other protocols. Figure 17 shows the average life cycle of protocols.

As shown in Figure 17, in random distribution the DCPVP protocol behaves better than other protocols for all cases. Also when the number of nodes increases, the DCPVP shows fewer downfalls in comparison with other algorithms. In Figure 17 as the number of nodes increases, the network lifetime would increase too, but when the number of nodes exceeds 150 nodes, the lifetime starts to decrease. This happens due to the structure of clusters and it seems that for this network topology, area dimensions and BS location, the optimum number of nodes is 150. For more nodes, the hot nodes which communicate directly to the BS, would be overloaded and the efficiency of the topology would decrease. The decline in lifetime is relative to the clustering structure and the selection progress of cluster heads. In addition, to provide a fair comparison between protocols, the load balance factor (LBF) is used, which was introduced and used in [24,35], respectively. As the cluster heads support its members and also route the data packets from the nodes belonging to other clusters, therefore, it is not desirable to have some overloaded cluster heads while some others are lightly loaded. At the same time, it is difficult to maintain a perfectly load balanced system during all times due to frequent detachment and attachment of the nodes from and to the CHs. To quantitatively measure how well balanced the cluster heads are, the authors in [24] introduced the LBF parameter. A higher value of LBF means better load balancing where is calculated as follows:

(13) $$LBF=\frac{N_{CH}}{\sum_{i=1}^{N_{CH}}{\left(-u\right)}^{2}}$$

where $N_{CH}$ is the number of CHs, members(i) is the number of members in cluster i and u can be calculated as follows:

(14) $$u=\frac{N-N_{CH}}{N_{CH}}$$

where N is the number of nodes and $N_{CH}$ is the number of clusters. The LBF is calculated for 100 nodes in random distribution and the results are shown in Table 4. Each value in Table 4 is the average of 10 experiments. As shown in Table 4, the DCPVP protocol has a higher LBF value that confirms the network lifetime extension.

**Table 4:** The Load Balance Factor for all protocols.

Protocol	LBF
LEACH	0.282
TCAC	0.374
HEED	0.301
WCA	0.324
GCMRA	0.386
DCPVP	0.422

## 6. Conclusions

This paper has proposed a new distributed clustering protocol using voting and priority ideas for virtual portioning sensor networks called the DCPVP protocol. The DCPVP method is an energy-efficient, scalable, load-balanced and self-organized clustering protocol that could be employed in large-scale and harsh environments. The size of clusters vary depending on the distances from the data link to overcome the energy hole problem for nodes closer to the base station. The results confirmed that DCPVP prolongs the network lifetime for both evenly and random sensor node distributions compared to some well-known clustering protocols in the literature such as the LEACH, HEED, WCA, GCMRA and TCAC protocols. The LBF values confirmed that the scalability of the DCPVP protocol enhances the lifetime of distributed sensor networks.
